# Isolation of C_11_ Cyclopentenones from Two Didemnid Species, *Lissoclinum* sp. and *Diplosoma* sp.

**DOI:** 10.3390/md7040816

**Published:** 2009-12-17

**Authors:** Takayuki Ogi, Palupi Margiastuti, Toshiaki Teruya, Junsei Taira, Kiyotake Suenaga, Katsuhiro Ueda

**Affiliations:** 1 Department of Chemistry, Biology and Marine Science, University of the Ryukyus, 1 Senbaru, Nishihara, Okinawa 903-0213, Japan; E-Mail: ogitkyuk@pref.okinawa.lg.jp (T.O.); 2 Okinawa Industrial Technology Center, 12-2 Suzaki, Uruma, Okinawa 904-2234, Japan; 3 Faculty of Education, University of the Ryukyus, 1 Senbaru, Nishihara, Okinawa 903-0213, Japan; E-Mail: teruya@edu.u-ryukyu.ac.jp (T.T.); 4 Department of Bioresources Engineering, Okinawa National College of Technology, 905 Henoko, Nago, Okinawa 905-2192, Japan; E-Mail: taira@okinawa-ct.ac.jp (J.T.); 5 Department of Chemistry, Keio University, 3-14-1 Hiyoshi, Kohoku-ku, Yokohama, Kanagawa 223-8522, Japan; E-Mail: suenaga@chem.keio.ac.jp (K.S.)

**Keywords:** Lissoclinum, Diplosoma, cyclopentenone, didemnenone, cytotoxicity

## Abstract

A series of new C_11_ cyclopentenones **1**–**7** was isolated, together with four known metabolites **9**/**10**, **12** and **13**, from the extract of the didemnid ascidian *Lissoclinum* sp. The other didemnid ascidian *Diplosoma* sp. contained didemnenones **1**, **2** and **5**, and five known metabolites **8–12**. The structures of **1–7** were elucidated by spectroscopic analyses. Cytotoxicity of the isolated compounds was evaluated against three human cancer cell lines (HCT116, A431 and A549).

## 1. Introduction

**Figure 1.**
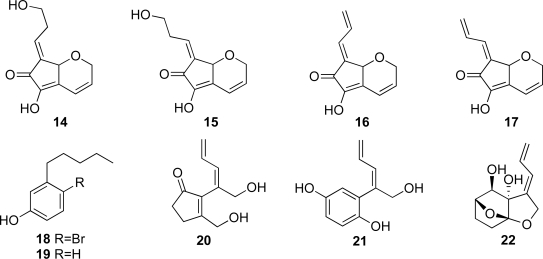
Some C_11_ metabolites isolated from marine organisms.

It has been amply demonstrated that ascidians are a prolific source of novel bioactive secondary metabolites [1–4]. Ascidians belonging to the family Didemnidae, e.g., *Lissoclinum* spp. and *Diplosoma* spp., harbor obligate cyanobacterial symbionts of the genus *Prochloron* [5–7], and have yielded structurally unique and pharmacologically interesting compounds such as patellazoles, varacin, virenamides, haterumalide and haterumaimides [8–15]. A series of C_11_ compounds having the distinctive exo-allylidene-lactone named didemnenone was isolated from didemnid ascidians, *Trididemnum cyanophorum* (didemnenones A and B) and *Didemnum voeltzkowi* (didemnenones C and D) [16]. They showed a wide range of biological activities, including cytotoxicity against leukemia cells as well as antimicrobial and antifungal activities [16–18]. Their structures were determined based on an X-ray investigation of the methylacetal of didemnenone A and from synthetic results [16–18]. As described previously, as part of our ongoing research aiming at the isolation of biologically active metabolites from marine organisms living in the tidal zone, we have isolated several C_11_ compounds, dinemnenone congeners **14**–**17** [19–22] and pentylphenols **18** and **19** [22] from ascidian *Diplosoma* spp. (Figure 1).

**Figure 2.**
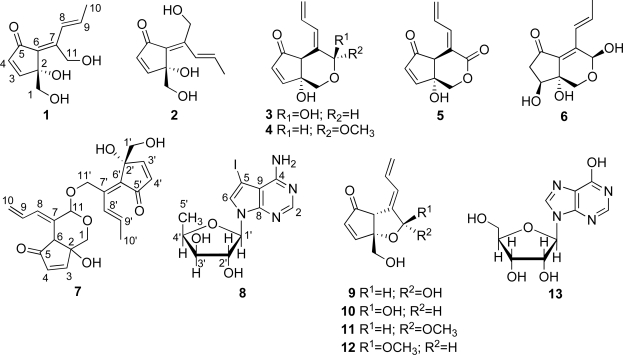
Compounds isolated from two didemnid ascidians.

Recently, we examined the constituents of ascidians, *Lissoclinum* sp. collected on the coast of Tarama island and *Diplosoma* sp. from dead corals of Hateruma island. From the *Lissoclinum* sp. we identified the new metabolites **1**–**7**, along with the known metabolites, didemnenones A (**9**) and B (**10**) as an inseparable mixture, a methylacetal of didemnenone B (**12**) [16], and inosine (**13**) (Figure 2). The *Diplosoma* sp. contained didemnenones **1**, **2** and **5** along with five known metabolites **8**–**12** (Figure 2) [16,23–34]. In this report, we describe the isolation, structure elucidation and bioactivity of these metabolites, and we also discuss the biosynthesis of didemnenones and the related compounds.

## 2. Results and Discussion

Specimens of colonial ascidian *Lissoclinum* sp. were collected off the coast of Tarama island, Okinawa, Japan. The specimens were extracted with acetone and the extract was partitioned between EtOAc and H_2_O. The aqueous layer was further partitioned between 1-BuOH and H_2_O. The H_2_O-soluble part was fractionated using RP-MPLC (reversed-phase MPLC) and eluted with a combination of H_2_O and MeOH. Further purification of the obtained fractions using RP-HPLC led to isolation of **1** (0.0059% of wet weight) and **13** (0.00067%). The EtOAc extract was suspended in aqueous MeOH and then successively extracted with hexane, CHCl_3_ and 1-BuOH. The resultant BuOH-soluble material was separated by a series of chromatographic steps [RP-OCC (reversed-phase open column chromatography), RP-MPLC and RP-HPLC] to afford **3** (0.0020%), **4** (0.00058%), **6** (0.0029%), **2** (0.011%), **1** (0.0049%), **12** (0.000014%), **5** (0.00030%), **7** (0.000019%) and an inseparable mixture of didemnenones A/B (**9**/**10**, 1:1, 0.00033%).

The colonial ascidian *Diplosoma* sp. was collected off the coast of Hateruma island, Okinawa, Japan. The specimens were extracted with acetone and the extract was partitioned between EtOAc and H_2_O. The EtOAc extract was suspended in aqueous MeOH and then successively extracted with hexane, CHCl_3_ and 1-BuOH. The CHCl_3_-soluble material was subjected to RP-OCC and eluted with H_2_O/MeOH, MeOH and MeOH/EtOAc. Further separation of the H_2_O/MeOH fraction by RP-HPLC eluted with a combination of H_2_O, MeOH and MeCN, led to the isolation of **1** (0.0021%), **2** (0.00066%), **5** (0.00047%), **8** (0.018%), **9**/**10** (1:1 mixture, 0.00069%), **11** (0.00098%) and **12** (0.00033%).

**Figure 3.**
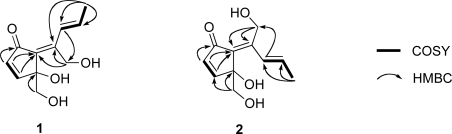
Partial structures of **1** and **2** based on COSY (bold line) and some important HMBC–correlations (arrows).

**Figure 4.**
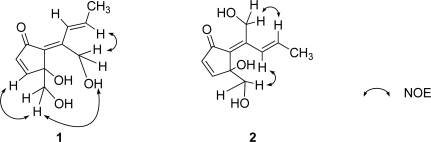
Selected NOEs for **1** and **2**.

Analysis of **1** by NMR (Tables 1 and 2) and HR-ESIMS [*m/z* 233.0786 (M + Na)^+^, calcd. for C_11_H_14_O_4_Na, 233.0784] provided a molecular formula of C_11_H_14_O_4_. The carbon resonating at δ_C_ 197.1 (s) suggested the presence of a carbonyl carbon in **1** and the IR absorption band at *v*_max_ 1675 cm^−1^ further supported the presence of the carbonyl group. Extensive analysis of ^1^H- and ^13^C-NMR data (Tables 1 and 2) supported by ^1^H-^1^H COSY data indicated the presence of a *cis* double bond [δ_C_ 161.4 (d), 134.3 (d); δ_H_ 7.35 (d), 6.25 (d)], a *trans* double bond [δ_C_ 127.2 (d), 134.8 (d); δ_H_ 7.73 (dd, *J* = 1.6, 16.0 Hz), 6.43 (dq, *J* = 16.0, 6.8 Hz)], a tetrasubstituted double bond [δ_C_ 133.5 (s), 144.5 (s)], a methyl group [δ_C_ 19.1 (t); δ_H_ 1.83 (dd)], two oxygenated methylenes [δ_C_ 66.8 (t); δ_H_ 3.74 (dd), 3.51 (dd) and δ_C_ 56.6 (t); δ_H_ 4.64 (dd), 4.36 (dd)] and an oxygenated quaternary carbon [δ_C_ 80.5 (s)] in **1**. Degrees of unsaturation for these partial structures amount to four. Thus, **1** must be monocyclic to account for the five degrees of unsaturation required by the molecular formula. The connectivity of the aforementioned partial structures was established from the HMBC correlations of H_2_-1/C-2, H-3/C-6, H-4/C-5, H-4/C-6, H-8/C-11, H_3_-10/C-7, H_3_-10/C-8, H_3_-10/C-9, H_2_-11/C-6 and H_2_-11/C-7, as shown in Figure 3, to describe the entire carbon framework of **1**. Geometric configuration of two olefins in **1** at C-6/C-7 and C-8/C-9 were assigned to be *E* by NOEDS experiments (Figure 4), in which irradiation of H-9 caused enhancement of H-11 and irradiation of H-1 resulted in enhancement of the H-3 and OH-11 proton signals. Therefore, the planar structure of **1** was established as a class of didemnenone as shown in **1**.

Compound **2** had the same molecular formula as **1**, C_11_H_14_O_4_, as established by HR-ESIMS [*m/z* 233.0786 (M + Na)^+^, calcd. for C_11_H_14_O_4_Na, 233.0784]. The IR absorption bands at *v*_max_ 1695 and 3360 cm^−1^ indicated the presence of carbonyl and hydroxyl groups. The NMR data (Tables 1 and 2) of **2** showed close similarity to those of **1**, except for chemical shifts of H-8. The chemical shift of H-8 in **2** (δ_H_ 6.91) was at higher field than in **1** (δ_H_ 7.73) due to the magnetic anisotropy effect of the carbonyl group, suggesting a *Z* configuration for the C-6 olefin in **2**. This was confirmed by NOEDS experiments (Figure 4), in which irradiation of H-9 caused enhancement of H-11 and irradiation of H-1 resulted in enhancement of H-8.

Analysis of the ^13^C-NMR and HR-ESIMS [*m/z* 231.0632, (M + Na)^+^, calcd for C_11_H_12_O_4_Na, 231.0628] for **3** provided a molecular formula C_11_H_12_O_4_, which suggested six degrees of unsaturation. The carbon resonating at δ_C_ 203.8 (s) suggested the presence of a carbonyl carbon in **3** and the IR absorption band at *v*_max_ 1710 cm^−1^ further supported the presence of the carbonyl group. The ^1^H- and ^13^C-NMR analyses (Tables 1 and 2) coupled with ^1^H-^1^H COSY data indicated the presence of an α, β-unsaturated ketone moiety [δ_C_ 203.8 (s), 132.8 (d), 165.1 (d); δ_H_ 6.28 (d), 7.52 (d)], a conjugated diene moiety [δ_C_ 135.0 (s), 131.7 (d); δ_H_ 6.48 (d) and δ_C_ 132.3 (d), 119.9 (t); δ_H_ 6.58 (ddd), 5.23 (d), 5.38 (d)], an acetal [δ_C_ 91.7 (d); δ_H_ 5.10 (brd)], a methine [δ_C_ 55.5 (d); δ_H_ 3.45 (s)], an oxygenated methylene [δ_C_ 63.2 (t); δ_H_ 3.53 (d), 3.42 (d)] and an oxygenated quaternary carbon [δ_C_ 79.7 (s)] in **3**. Extensive analysis of ^1^H-^1^H COSY demonstrated two isolated spin systems, C-3–C-4 and C-8–C-10. The connectivity of the aforementioned partial structures was established from the HMBC correlations of H_2_-1/C-2, H_2_-1/C-3, H_2_-1/C-6, H_2_-1/C-11, H-3/C-2, H-3/C-4, H-3/C-5, H-3/C-6, H-6/C-2, H-6/C-4, H-6/C-5, H-6/C-7, H-6/C-11, H-8/C-6, H-8/C-11, H-9/C-7, H-11/C-1, H-11/C-6 and H-11/C-7, to describe the entire carbon framework of **3**. Geometric configuration of the olefins in **3** at C-7/C-8 was assigned to be *E* by NOEDS experiments, in which irradiation of H-6 caused enhancement of H-9 and irradiation of H-8 caused enhancement of H-11 and H-10. Therefore, the planar structure of **3** was concluded to be a class of didemnenone, as depicted in **3**. The NOE observed between a hydroxyl proton (OH-2) and H-6 revealed a *cis* fusion of two rings. The NOEs; OH-2/H-11; H-1b/H-11 allowed the assignment of the H-11 as α.

The molecular formula of **4** was deduced to be C_12_H_14_O_4_ based on HR-ESIMS [*m/z* 245.0794, (M + Na)^+^, calcd for C_12_H_14_O_4_Na, 245.0784]. The ^1^H- and ^13^C-NMR spectral data (Tables 1 and 2) of **4** resembled those of **3**, except for the presence of a proton signal at δ_H_ 3.31 (s) and a carbon signal at δ_C_ 54.9 (q) in **4**. Geometric configuration of the double bond at C-7/C-8 in **4** was assigned to be *E* by NOEDS experiments, in which irradiation of H-6 caused enhancement of H-9 and irradiation of H-8 caused enhancement of H-11 and H-10. Therefore, the planar structure of **4** was elucidated to be a methylacetal of **3**. The NOE between a hydroxyl proton (OH-2) and H-6 also indicated the ring junction to have the *cis*-geometry. The NOEs; OH-2/ H-1b; H-1a /H-11 allowed the assignment of the H-11 as β. We cannot affirm that **4** is a natural product, because it is conceivable it arises from **3** in the isolation process.

The molecular formula of **5** was determined to be C_11_H_10_O_4_, based on HR-ESIMS [*m/z* 207.0654 (M + H)^+^, calcd for C_11_H_11_O_4_, 207.0652]. The carbons resonating at δ_C_ 202.9 (s) and 166.9 (s) suggested the presence of a carbonyl carbon and an ester carbonyl carbon, respectively in **5** (Table 2) and the IR absorption band at *v*_max_ 1717 cm^−1^ further supported the presence of the carbonyl groups. The ^1^H- and ^13^C-NMR (Tables 1 and 2) and 2D NMR spectral data of **5** are similar to those of γ-lactone didemnenone [16], except for the HMBC correlation observed between an oxymethylene proton H_2_-1 at δ_H_ 4.13 and a carbonyl carbon C-11 at δ_C_ 166.9. Geometric configuration of the double bond at C-7/C-8 in **5** was assigned to be *E* by NOEDS experiments, in which irradiation of H-6 caused enhancement of H-9. Therefore, the planar structure of **5** was concluded to be a class of didemnenone, as depicted in **5**.The NOE between a hydroxyl proton (OH-2) and H-6 allowed the ring junction to be assigned as *cis*.

Analysis of the ^13^C-NMR and HR-ESIMS [*m/z* 249.0742, (M + Na)^+^, calcd for C_11_H_14_O_5_Na, 249.0733] for **6** provided a molecular formula C_11_H_14_O_5_. The carbon resonating at δ_C_ 206.1 (s) suggested the presence of a carbonyl carbon in **6** (Table 2) and the IR absorption band at *v*_max_ 1720 cm^−1^ further supported the presence of the carbonyl group. Extensive analysis of ^1^H- and ^13^C-NMR data (Tables 1 and 2) supported with ^1^H-^1^H COSY data indicated the presence of a *trans* double bond [δ_C_ 124.4 (d), 135.3 (d); δ_H_ 7.27 (dd, *J* = 1.4, 16.0 Hz), 6.31 (dq, *J* = 16.0, 6.8 Hz)], a tetrasubstituted double bond [δ_C_ 130.4 (s), 142.3 (s)], an oxygenated methylene [δ_C_ 63.1 (t); δ_H_ 4.27 (d), 3.50 (d)], two oxygenated methines [δ_C_ 86.6 (d); δ_H_ 5.51 (d) and δ_C_ 70.5 (d); δ_H_ 3.99 (brt)], an oxygenated quaternary carbon [δ_C_ 73.0 (s)], a methylene [δ_C_ 46.3 (t); δ_H_ 2.74 (dd), 1.98 (brd)] and a methyl [δ_C_ 19.1 (q); δ_H_ 1.82 (dd)] in **6**. These functionalities accounted for three of the five degrees of unsaturation, therefore **6** is bicyclic. The connectivity of the aforementioned partial structures was established from the HMBC correlations of H-1/C-2, H-1/C-6, H-1/C-11, H-4/C-5, H-4/C-6, H-8/C-11 and H-11/C-6, to describe the entire carbon framework of **6**. The NOE between H-9 and H-11 also indicated the double bond at C-8 to have the *trans-*geometry, and the NOEs; OH-2/H-1a; OH-2/H-3; H-1a/H-11 allowed the assignment of the 3-OH and the 11-OH both as β.

Analysis of **7** by ^13^C-NMR (Table 3) and HR-ESIMS [*m/z* 401.1602 (M + H)^+^, calcd for C_22_H_25_O_7_, 401.1595] provided a molecular formula of C_22_H_24_O_7_. Extensive analysis of ^1^H- (Table 3) and ^13^C-NMR data, supported with ^1^H-^1^H COSY data, indicated the presence of two α, β-unsaturated ketone moieties [δ_C_ 203.6 (s), 132.8 (d), 165.1 (d); δ_H_ 6.31 (d), 7.55 (d) and δ_C_ 196.7 (s), 134.9 (d), 161.6 (d); δ_H_ 6.28 (d), 7.37(d)], a conjugated diene moiety [δ_C_ 132.6 (s), 132.8 (d); δ_H_ 6.37 (d) and δ_C_ 132.2 (d), 120.3 (t); δ_H_ 6.59 (ddd), 5.30 (d), 5.26 (d)], a *trans* double bond [δ_C_ 126.9 (d), 134.7 (d); G_H_ 7.78 (dd, *J* = 1.4, 16.0 Hz), 6.41 (m)], a tetrasubstituted double bond [δ_C_ 135.2 (s), 140.7 (s)], an acetal [δ_C_ 97.9 (d); δ_H_ 5.08 (s)], three oxygenated methylenes [δ_C_ 63.3 (t); δ_H_ 3.53 (brd), δ_C_ 66.6 (t); δ_H_ 3.70 (dd), 3.46 (dd) and δ_C_ 62.7 (t); δ_H_ 4.86 (d), 4.49 (d)], two oxygenated quaternary carbons [δ_C_ 79.1 (s), 80.4 (s)], a methine [δ_C_ 55.1 (d); δ_H_ 3.48 (s)] and a methyl [δ_C_ 19.1 (q); δ_H_ 1.88 (dd)] in **7**. The structure of **7** was elucidated to be a dimeric didemnenone composed of **1** and **3**, from the molecular formula, the NMR data and the HMBC correlations of H-11/C-11′ and H_2_-11′/C-11. Geometric configuration of three olefins in **7** at C-7/C-8, C-6′/C-7′ and C-8′/C-9′ was assigned to be *E* by NOEDS experiments, in which irradiation of H-6 caused enhancement of H-9, irradiation of H-8 caused enhancement of H-11 and H-10, irradiation of H-9′ caused enhancement of H-11′ and irradiation of H-3′ resulted in enhancement of the H-1′ proton signal. Therefore, the structure of **7** was established as a didemnenone dimer as shown in **7**. We could not determine the C-2/C-6 ring junction stereochemistry owing to decomposition of **7**.

**Figure 5.**
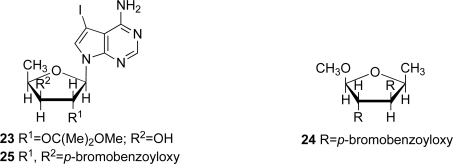
Compounds used for determining the absolute structure of **8**.

The structure of marine metabolite **8** was determined to be 4-amino-7-(5’-deoxy-β-d-xylofuranosyl) -5-iodopyrrolo[2,3-*d*]pyrimidine by 1D and 2D NMR spectra for **8** and **23**, and by CD spectra of compounds **8**, **24** and **25** (Figure 5), as previously described [23]. The absolute stereochemistry of the new compounds was tentatively deduced to be as depicted in **1**–**7** based on the assumption that there is a similar biogenetic relationship between these compounds and (+)-didemnenone A. (+)Didemnenones **9**–**12** and inosine (**13**) were unambiguously identified by comparison of their spectral data with those described in the literature [16].

Compounds **1–13** were tested *in vitro* for their cytotoxic activities against the HCT116, A431 and A549 cancer cell lines (Table 4). Compounds **1**, **2** and **8** were significantly cytotoxic against the HCT116, A431 and A549 cancer cell lines, and compounds **3**, **4**, **7**, **9**/**10** and **12** were significantly cytotoxic against two cell lines, HCT116 and A431. In contrast to **12** (a β-anomer), its isomer **11** (an α-anomer) was not cytotoxic against any of the three cell lines. Among the isolated compounds tested, the iodinated nucleoside **8** showed the strongest cytotoxic activity against the HCT116, A431 and A549 cancer cell lines, with IC_50_ values of 1.8, 3.1 and 3.5 μ g/mL, respectively.

To date, a variety of C_11_ compounds have been isolated from ascidians (compounds **9** and **10)**, cyanobacteria (compounds **20** and **21**) and a sponge (compound **22**) (Figures 1 and 2) [16,35,36]. Compounds **16** and **17** have been isolated from the ascidian *Diplosoma virens* and a sponge *Ulosa* sp. (Figure 2) [19,20]. Isolation of a series of the C_11_ compounds including compounds **18** and **19** (Figure 2) from unrelated marine organisms supports the potential microbial origin of these compounds. From this viewpoint, we assume that the ascidian *Diplosoma* sp. might not be the actual producer of the C_11_ compounds, but suggest a possible microorganism source such as *Prochloron* spp. We conducted, therefore, the following experiments. The *Prochloron* spp., which are obligatory symbionts of ascidians, were separated from the body of the ascidians *Lissoclinum* sp. and *Diplosoma* spp. by squeezing through the plankton net. ^1^H-NMR spectra of the acetone extracts of the separated *Prochloron* spp. showed the presence of the same peaks as present in those of didemnenones. This confirms our assumption that *Prochloron* spp. are the actual producers of didemnenones.

**Scheme 1.**
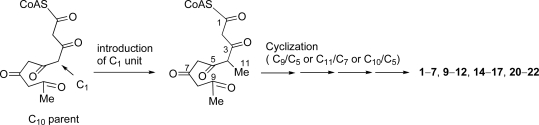
Plausible biosynthesis of the carbon skeletons for didemnenones and related compounds.

Most C_11_ compounds are derived from polyketides (six acetates-C_1_) or polyketides (five acetates + C_1_) [37]. Pentylphenols such as **18** and **19**, and some compounds which have a carbon skeleton of 5-methyldecane are known to be derived from the former with a loss of CO_2_ from C_12_ parent (six acetates). Some C_11_ metabolites are ascertained to be derived from a polyketide precursor (five acetates + C_1_) which has a carbon skeleton of 4-methyldecane [37]. We found that didemnenone-related compounds **1**–**7**, **9**–**12**, **14**–**17** and **20**–**22** have a common carbon skeleton of 4-methyldecane from a consideration of the carbon skeleton of these compounds. Consequently, We propose that these compounds should be derived from the polyketides (five acetates + C_1_) *via* cyclization between C-9/C-5, between C-11/C-7 or between C-10/C-5 (Scheme 1).

## 3. Experimental Section

### 3.1. General experimental procedures

Optical rotations were measured on either a JASCO P-1020 or JASCO DIP-1000 polarimeter. Ultraviolet-visible spectra were obtained in methanol on a JASCO V-550 spectrophotometer. Infrared spectra were recorded as dry films on either JASCO FT/IR-300 or Spectrum 2000 Explorer (PERKIN ELMER). CD spectra were recorded on a JASCO J-720W Circular Dichroism Spectrometer. ^1^H- and ^13^C-NMR spectra were recorded on a JEOL JNM α-500 FT-NMR spectrometer or a JEOL JNM lambda 400 FT-NMR spectrometer, and chemical shifts were referenced to the solvent signals [δ_H_ 7.24 and δ_C_ 77.0 in chloroform-*d*, δ_H_ 2.49 and δ_C_ 39.5 in DMSO-*d*_6_*,* δ_H_ 3.30 and δ_C_ 49.0 in methanol-*d*_4_]. Inversed-detected heteronuclear correlations were measured using HMQC and HMBC pulse sequences with a pulse field gradient. HR-ESIMS data were obtained on a LTQ ORBITRAP (ThermoFisher Scientific, Germany), and HR-FABMS data were obtained on a JEOL JMS-700 mass spectrometer. LR-ESIMS data were measured on a Waters *Quattro micro* API triple quadruple mass analyzer. RP-OCC and RP-MPLC were performed on COSMOSIL^®^ 140C_18_-OPN. Preparative RP-HPLC was run on a Waters 600 multi solvent system using ODS columns (YMC-Pack ODS-A, 150 × 20 mm I.D., YMC-Pack ODS-A, 250 × 20 mm I.D., YMC-Pack ODS-C8, 250 × 20 mm I.D., YMC-Pack ODS-AQ, 250 × 20 mm I.D., Develosil ODS-HG-5, 250 × 20 mm I.D. and COSMOSIL^®^ -packed C_18_, 250 × 10 mm I.D.). All solvents used were reagent grade.

### 3.2. Animal material

The colonial brown ascidian was collected by hand at the tidal zone of Tarama island, Okinawa, Japan, and the colonial green ascidian was collected by hand from the coast of Hateruma island, Okinawa, Japan. The ascidians were stored at −15 ^°^C until extraction. The brown ascidian and the green ascidian were identified as *Lissoclinum* sp. and *Diplosoma* sp., respectively, by Euichi Hirose, University of the Ryukyus, Japan. The voucher specimens were deposited at the University of the Ryukyus (Specimen no. URKU-801 for the brown ascidian and URKU-802).

### 3.3. Extraction and isolation

The ascidian *Lissoclinum* sp. (5.7 kg, wet weight) was initially extracted with acetone (18 L) and filtered to remove debris. The filtrate was concentrated *in vacuo* to remove acetone and the resultant mixture was partitioned between EtOAc (3.5 L) and H_2_O (3.5 L). The H_2_O layer was extracted with 1-BuOH to give the 1-BuOH extract (18.3 g) and the H_2_O extract (141.2 g). An aliquot of 27.8 g of the H_2_O-soluble material was subject to RP-MPLC on ODS (COSMOSIL^®^ 140C_18_-OPN, 140 μm, 50 × 3 cm I.D.) with H_2_O (500 mL), H_2_O/MeOH (9:1, 500 mL 7:3, 500 mL; 5:5, 500 mL, 3:7, 500 mL; 1:9, 500 mL) and MeOH (500 mL) to give seven fractions. The second fraction (860 mg) was subjected to HPLC on ODS [YMC-Pack ODS-AQ, 250 × 20 mm I.D.; linear gradient elution, H_2_O/MeOH (3:1)-MeOH] to give 18 fractions. The 18th fraction contained pure **1** (66.0 mg). The 14th fraction (84.0 mg) was subjected to HPLC on ODS [YMC-Pack ODS-AQ, 250 × 20 mm I.D.; linear gradient elution, 1% HCOOH /H_2_O (1:9)-1% HCOOH/MeCN (1:9)] to give **13** (7.6 mg). The EtOAc layer was concentrated *in vacuo* to give a brown material (29.3 g). The material was subjected to a modified Kupchan’s partitioning procedure as follows. The material was suspended in a mixture of H_2_O/MeOH (1:1) and then successively extracted with hexane and CHCl_3_. The resultant aqueous phase was concentrated to remove MeOH and then extracted with 1-BuOH. An aliquot of 0.58 g of the BuOH-soluble material (3.93 g) was separated by RP-MPLC (COSMOSIL^®^ 140C_18_-OPN, 140 μm, 50 × 3 cm I.D.) using H_2_O (500 mL), H_2_O/MeOH (9:1, 500 mL 7:3, 500 mL; 5:5, 500 mL, 3:7, 500 mL; 1:9, 500 mL) and MeOH (500 mL) to give seven fractions (fr. 1–7). The first fraction (fr. 1, 436 mg) was purified by RP-HPLC [YMC-Pack ODS-A, 250 × 20 mm I.D.; linear gradient elution, H_2_O/MeOH (4:1)-MeOH] to afford **3** (165 mg) and **4** (3.2 mg). The second fraction (fr. 2, 272 mg) was separated by RP-HPLC [YMC-Pack ODS-A, 150 × 20 mm I.D.; linear gradient elution, H_2_O/MeOH (4:1)-MeOH] to give 18 fractions (fr. 2-1–2-18). Purification of the second fraction (fr. 2-2, 170 mg) by RP-HPLC (Develosil ODS-HG-5, 250 × 20 mm I.D.) using H_2_O/MeOH (9:1) led to the isolation of **6** (24.6 mg), **2** (92.3 mg), **1** (92.7 mg) and an inseparable mixture of didemnenones A/B (**9/10**, 2.8 mg). HPLC separation [Develosil ODS-HG-5, 250 × 20 mm I.D., H_2_O/MeOH (9:1)] of seventh fraction (fr. 2–7, 6.8 mg) led to the isolation of **12** (0.8 mg). An aliquot of 3.35 g of the 1-BuOH extract (3.93 g) was chromatographed on ODS (COSMOSIL^®^ 140C_18_-OPN, 140 μm, 100 g) with H_2_O/MeOH (3:7, 500 mL; 2:8, 100 mL; 1:9, 100 mL) and MeOH (100 mL) to give nine fractions. The second fraction (1.1 g) was purified by HPLC on ODS [YMC-Pack C8, 250 × 20 mm I.D.; linear gradient elution, H_2_O/MeCN (8:2–1:9)], to give **1** (186.8 mg), **5** (14.6 mg), **4** (29.7 mg) and **7** (0.9 mg).

The ascidian *Diplosoma* sp. (900 g, wet weight) was initially extracted with acetone (2.2 L). After filtration, the extracts were concentrated in *vacuo* to give an acetone extract. The acetone extract was partitioned between EtOAc and H_2_O. The H_2_O layer was further extracted with 1-BuOH. The 1-BuOH layer was concentrated *in vacuo* to give a BuOH-soluble material (2.14 g). The EtOAc layer was concentrated *in vacuo* to give an EtOAc extract (7 g). The EtOAc extract was suspended in H_2_O/MeOH (1:1, 400 mL) and then successively extracted with hexane and CHCl_3_ to give a hexane-soluble material (4.0 g), a CHCl_3_-soluble material (2.0 g) and an aqueous material (2.1 g). The aqueous material and the BuOH-soluble material (2.14 g) were combined. The combined polar fraction (4.24 g) was chromatographed on ODS (COSMOSIL^®^ 140C_18_-OPN) with H_2_O/MeOH (1:1, 2:1), MeOH and MeOH/EtOAc (9:1) as eluent. The first fraction (2.0 g) contained more than 95% of **1** from its ^1^H NMR data. The CHCl_3_-soluble material (2.0 g) was subjected to OCC on ODS [COSMOSIL^®^ 140C_18_-OPN, H_2_O/MeOH (1:4), MeOH and MeOH/EtOAc (7:3)]. The first fraction (fr. 1, 1.0 g) was further separated by OCC on ODS (COSMOSIL^®^ 140C_18_-OPN) using H_2_O/MeOH (1:1, 2:1), MeOH and MeOH/EtOAc (9:1). The first aqueous methanol fraction (fr. 1-1, 902 mg) was subjected to HPLC on ODS (COSMOSIL^®^ -packed C_18_, 250 × 10 mm I.D.) using H_2_O/MeOH (1:1) to give six fractions (fr. 1-1-1-fr. 1-1-6). The first fraction (fr. 1-1-1, 92.9 mg) was separated by HPLC on ODS [COSMOSIL^®^ -packed C_18_, 250 × 10 mm I.D., H_2_O/MeCN (1:1)] first, and then by RP-HPLC [COSMOSIL^®^-packed C_18_, 250 × 10 mm I.D., H_2_O/MeOH/MeCN (5:2:2)] to yield **1** (18.7 mg) and **2** (5.9 mg). Further separation of the second fraction (fr. 1-1-2, 61.6 mg) by repeated HPLC [COSMOSIL^®^ -packed C_18_, 250 × 10 mm I.D., H_2_O/MeOH/MeCN (50:31:19) and then H_2_O/MeCN (1:1)] to give **5** (4.2 mg). An inseparable mixture (6.2 mg) of didemnenones A and B (**9** and **10**) was obtained along with their methylacetals **11** (8.8 mg) and **12** (3.0 mg) from the fourth fraction (fr. 1-1-4, 70.4 mg) by repeated ODS HPLC [COSMOSIL^®^ -packed C_18_, 250 × 10 mm I.D., H_2_O/MeOH/MeCN (50:31:19) and then H_2_O/MeCN (1:1)]. The fifth fraction (fr. 1-1-5, 40.0 mg) was purified by RP-HPLC (COSMOSIL^®^-packed C_18_, 250 × 10 mm I.D.) with H_2_O/MeOH/MeCN (50:31:19) to afford **8** (9.9 mg). The sixth fraction (fr. 1-1-6, 210.7 mg) was purified by HPLC on ODS (COSMOSIL^®^-packed C_18_, 250 × 10 mm I.D.) using MeOH/EtOAc (19:1) to yield **8** (164.4 mg).

#### 3.3.1. Compound **1**

Pale yellowish oil; [α]^27^_D_ +21.0° (*c* 0.66, MeOH); UV (MeOH) *λ*_max_ (log *ɛ*) 246 (3.39), 308 (3.69) nm; FT/IR (film) *v*_max_ 3355, 1675, 1035 cm^−1^; ^1^H-NMR and ^13^C-NMR (DMSO-*d*_6_) see Tables 1 and 2; LR-ESIMS *m/z* 211 (M + H)^+^, 209 (M − H)^−^ HR-ESIMS *m/z* (M + Na)^+^ 233.0786 (calcd. for C_11_H_14_O_4_Na, 233.0784).

#### 3.3.2. Compound **2**

Pale yellowish oil; [α]^27^_D_ +22.5° (*c* 0.43, MeOH); UV (MeOH) *λ*_max_ (log *ɛ*) 245 (3.38), 307 (3.74) nm; FT/IR (film) *v*_max_ 3359, 1712, 1379 cm^−1^; ^1^H-NMR and ^13^C-NMR (DMSO-*d*_6_) see Tables 1 and 2; LR-ESIMS *m/z* 233 (M + Na)^+^, 211 (M + H)^+^, 209 (M − H)^−^; HR-ESIMS *m/z* (M + Na)^+^ 233.0786 (calcd. for C_11_H_14_O_4_Na, 233.0784).

#### 3.3.3. Compound **3**

Pale yellowish oil; [α]^24^_D_ +147° (*c* 0.62, H_2_O); UV (H_2_O) *λ*_max_ (log *ɛ*) 246 (3.51), 274 (3.54); FT/IR (film) *v*_max_ 3400, 1710, 1050 cm^−1^; ^1^H-NMR and ^13^C-NMR (DMSO-*d*_6_) see Tables 1 and 2; LR-ESIMS *m/z* 231 (M + Na)^+^, 207 (M − H)^−^; HR-ESIMS *m/z* (M + Na)^+^ 231.0632 (calcd. for C_11_H_12_O_4_Na, 231.0628).

#### 3.3.4. Compound **4**

Pale yellowish oil; [α]^24^_D_ +459° (*c* 0.015, MeOH); UV (MeOH) *λ*_max_ (log *ɛ*) 249 (3.65); FT/IR (film) *v*_max_ 3410, 1700, 1045 cm^−1^; ^1^H-NMR and ^13^C-NMR (DMSO-*d*_6_) see Tables 1 and 2; LR-ESIMS *m/z* 223 (M + H)^+^; HR-ESIMS *m/z* (M + Na)^+^ 245.0794 (calcd. for C_12_H_14_O_4_Na, 245.0784).

#### 3.3.5. Compound **5**

Pale yellowish oil; [α]^27^_D_ + 112° (*c* 0.26, MeOH); UV (MeOH) *λ*_max_ (log *ɛ*) 273 (3.83); FT/IR (film) *v*_max_ 3419, 1717, 1049 cm^−1^; ^1^H-NMR and ^13^C-NMR (DMSO-*d*_6_) see Tables 1 and 2; LR-ESIMS *m/z* 207 (M + H)^+^, 205 (M − H)^−^; HR-ESIMS *m/z* (M + H)^+^ 207.0654 (calcd. for C_11_H_11_O_4_, 207.0652).

#### 3.3.6. Compound **6**

Pale yellowish oil; [α]^24^_D_ +16.3° (*c* 0.17, MeOH); UV (MeOH) *λ*_max_ (log *ɛ*) 294 (3.91); FT/IR (film) *v*_max_ 3390, 1720, 1050 cm^−1^; ^1^H-NMR and ^13^C-NMR (DMSO-*d*_6_) see Tables 1 and 2; LR-ESIMS *m/z* 249 (M + H)^+^, 223 (M + H)^+^; HR-ESIMS *m/z* (M + Na)^+^ 249.0742 (calcd. for C_11_H_14_O_5_Na, 249.0733).

#### 3.3.7. Compound **7**

Pale yellowish oil; [λ]^24^_D_ +11.7° (*c* 0.12, MeOH); UV (MeOH) *λ*_max_ (log *ɛ*) 248 (3.78), 308 (3.83); FT/IR (film) *v*_max_ 3400, 1580, 1050 cm^−1^; ^1^H-NMR and ^13^C-NMR (DMSO-*d*_6_) see Table 3; LR-ESIMS *m/z* 423 (M + Na)^+^, 401 (M + H)^+^, 399 (M − H)^−^; HR-ESIMS *m/z* (M + Na)^+^ 423.1423 (calcd for C_22_H_24_O_7_Na, 423.1414), (M + H)^+^ 401.1602 (calcd. for C_22_H_24_O_7_, 401.1595).

#### 3.3.8. Compound **8**

Pare yellowish oil: [α]^26^_D_ −69° (*c* 0.1, MeOH); UV (MeOH) *λ*_max_ (log *ɛ*) 283 nm (3.59); FT/IR (film) *v*_max_ 3461, 3317, 3132, 1633, 1584, 1474, 1084, 755 cm^−1^; NMR data were described in the previous paper [13]. LR-EIMS *m/z* (rel.%) 376 (M^+^, 7), 303 (3), 289 (13), 261 (33), 260 (100), 233 (18). HR-FABMS *m/z* (M)^+^ 376.0016 (calcd. for C_11_H_13_IN_4_O_3_, 376.0027).

#### 3.3.9. Acetal **23**

To a solution of iodinated nucleoside **8**, (10.0 mg, 26.6 μmol) in 2,2-dimethoxypropane (1 mL) and acetone (2 mL) was added a catalytic amount of camphorsulfonic acid. The mixture was stirred at rt for 24 h and at 45 °C for 4 h. The reaction mixture was diluted with ether, washed with saturated aqueous Na_2_CO_3_ and brine. The organic phase was dried (MgSO_4_) and concentrated *in vacuo*. The residual oil was purified by preparative TLC [CHCl_3_-MeOH (3:0.2)] to give the acetal as colorless oil (**23**, 3.0 mg, 25%). HR-FABMS and ^1^H NMR data for compound **23** were described in the earlier paper [23].

#### 3.3.10. Methyl 5-deoxy 2.3-di-O-(4-bromobenzoyl)-β-l-xylofuranoside (**24**)

The mixture of methyl 5-deoxy-β-l-xylofuranoside [38,39] (56.5 mg, 0.11 mmol), 4-bromobenzoyl chloride (250.1 mg, 0.87 mmol), and DMAP (2.2 mg, 0.018 mmol), in pyridine (0.8 mL) was stirred at ambient temperature for 24 h, and water (0.5 mL) was added to the mixture. After being stirred at ambient temperature for 30 min, the mixture was concentrated. The residual solid was purified by HPLC [Develosil ODS-HG-5 (250 × 20 mm I.D.); flow rate 5 mL/min; detection UV 256 nm; solvent 85% MeOH] to give methyl 5-deoxy 2.3-di-*O*-(4-bromobenzoyl)-β-l-xylofuranoside (**24**, 17.2 mg, 30%) and its corresponding α-anomer (15.3 mg, 27%), respectively. Spectral data for compound **24** were described in the previous paper [23].

#### 3.3.11. Dibenzoate **25**

The mixture of iodinated nucleoside **8** (5.0 mg, 0.013 mmol), 4-bromobenzoyl chloride (25.2 mg, 0.115 mmol), and DMAP (2.2 mg, 0.018 mmol), in pyridine (0.5 mL) was stirred at ambient temperature for 24 h, and water (0.5 mL) was added to the mixture. After being stirred at ambient temperature for 30 min, the mixture was concentrated. The residual solid was purified by HPLC [Develosil ODS-HG-5 (250 × 20 mm I.D.); flow rate 5 mL/min; detection UV 256 nm; solvent 85% MeOH] to give dibenzoate **25** (2.0 mg, 21%). Spectral data for compound **25** were described in the earlier paper [23].

#### 3.3.12. Condition of cell cultures

Human colorectal carcinoma (HCT116), human epidermal carcinoma (A431) and human lung cancer (A549) cells were cultured in DMEM medium (including 10% FBS, 100 U/mL penicillin and 100 ng/mL streptomycin) at 37 °C in a 5% CO_2_ atmosphere.

#### 3.3.13. Determination of cytotoxicity

Growth inhibition experiments were carried out in quadruplicate on 96-well flat-bottomed microplates, and the amount of viable cells at the end of incubation was determined with the MTT [3-(4,5-dimethylthiazol-2-yl) 2,5-diphenyltetrazolium bromide] dye reduction assay. Test compounds were dissolved in DMSO (4.0 mg/mL) and diluted with H_2_O such that the final DMSO concentration was 0.5%. Viable cells (HCT116, A431 and A549) in the growth medium were seeded on 96-well microplates (1.0 × 10^4^ cells/well) and incubated at 37 °C in a 5% CO_2_ atmosphere, and continuously cultured without or with five concentrations (20, 10, 5, 2.5, 1.25 μg/mL, final concentration) of test compounds for 48 h from the next day. After incubation, 10 μL of MTT (5 mg/mL in phosphate-buffer saline) was added each well, the samples were again incubated. After standing for 3 h, the medium was removed, and the resulting formazan crystals were dissolved with DMSO (100 μL). The optical density (O.D.) was measured at 570 nm, provided the reference for reading at 655 nm with a microplate reader (Model 550, BIO-RAD, USA).

## 4. Conclusions

We have isolated seven new didemnenone derivatives **1**–**7** together with four known metabolites **9**/**10**, **12** and **13** from *Lissoclinum* sp. We have also isolated didemnenones **1**, **2** and **5** and five known metabolites **8**–**12** from the other didemnid ascidian *Diplosoma* sp. Compounds **1**–**4**, **7**–**10** and **12** were significantly cytotoxic against the HCT116, A431 and/or A549 cancer cell lines. Didemnenone-related compounds **1**–**7**, **9**–**12**, **14**–**17** and **20**–**22**, which have been ever isolated from marine organisms [19–22,35,36], were found to have a common carbon skeleton of 4-methyldecane. From consideration of the carbon skeleton of these compounds, a biosynthesis of the carbon skeletons for didemnenones and related compounds was proposed (Scheme 1). ^1^H-NMR spectra of the extracts of the separated *Prochloron* spp. from the body of the ascidians *Lissoclinum* sp. and *Diplosoma* spp.showed the presence of the same peaks as present in those of didemnenones, which suggested that *Prochloron* spp. are the actual producers of didemnenones.

## Figures and Tables

**Table 1.** ^1^H-NMR data for compounds **1**–**6**.

**Pos.**	**δ_H_ (mult., *J*/Hz)^a^**					
	**1**	**2**	**3**	**4**	**5**	**6**
1a	3.74 (dd, 6.0, 11.2)	3.68 (dd, 6.2, 10.8)	3.53 (d, 11.4)	3.49 (d, 11.2)	4.26 (d, 11.2)	4.27 (d, 11.4)
1b	3.51 (dd, 6.0, 11.2)	3.49 (dd, 6.2, 10.8)	3.42 (d, 11.4)	3.43 (d, 11.2)	4.13 (d, 11.2)	3.50 (d, 11.4)
3	7.35 (d, 6.0)	7.35 (d, 6.2)	7.52 (d, 5.6)	7.52 (d, 5.6)	7.71 (d, 5.6)	3.99 (brt, 4.8)
4a	6.25 (d, 6.0)	6.27 (d, 6.2)	6.28 (d, 5.6)	6.30 (d, 5.6)	6.40 (d, 5.6)	2.74 (dd, 4.8, 18.4)
4b						1.98 (brd, 18.4)
6			3.45 (s)	3.48 (s)	3.74 (s)	
8	7.73 (dd, 1.6, 16.0)	6.91 (d, 16.0)	6.48 (d, 11.2)	6.45 (brd, 10.8)	7.22 (d, 11.6)	7.27 (dd, 1.4, 16.0)
9	6.43 (dq, 16.0, 6.8)	6.43 (dq, 16.0, 5.8)	6.58 (ddd, 11.2,	6.59 (ddd, 10.4,	6.86 (ddd, 10.0,	6.31 (dq, 16.0, 6.8)
			11.6, 16.4)	10.8, 16.4)	11.6, 16.8)	
10a	1.83 (dd, 1.6, 6.8)	1.85 (d, 5.8)	5.38 (d, 16.4)	5.40 (brd, 2.0, 16.4)	5.85 (dd, 1.6, 10.0)	1.82 (dd, 1.4, 6.8)
10b			5.23 (d, 11.6)	5.26 (brd, 2.0, 10.0)	5.67 (dd, 1.6, 16.8)	
11a	4.64 (dd, 6.8, 13.4)	4.82 (dd, 5.6, 11.2)	5.10 (brd, 5.8)	4.82 (s)		5.51 (d, 5.4)
11b	4.36 (dd, 6.8, 13.4)	4.59 (dd, 5.6, 11.2)				
OH-1	4.89 (t, 6.0)	4.84 (t, 6.2)				
OH-2	5.72 (s)	5.73 (s)	5.87 (s)	5.94 (s)	6.33 (brs)	5.24 (s)
OH-3						5.14 (brd, 4.8)
OH-11	4.66 (brs)	4.53 (t, 5.6)	6.73 (d, 5.8)			6.59 (d, 5.4)
OCH_3_				3.31 (s)		

a Recorded at 400 MHz in DMSO-d6.

**Table 2.** ^13^C-NMR data for compounds **1–6**.

**δ_C_ (mult.)^a^**						
**C no.**	**1**	**2**	**3**	**4**	**5**	**6**
1	66.8 (CH_2_)	66.3 (CH_2_)	63.2 (CH_2_)	63.1 (CH_2_)	69.7 (CH_2_)	63.1 (CH_2_)
2	80.5 (qC)	80.4 (qC)	79.7 (qC)	79.2 (qC)	75.8 (qC)	73.0 (qC)
3	161.4 (CH)	161.4 (CH)	165.1 (CH)	165.1 (CH)	164.9 (CH)	70.5 (CH)
4	134.3 (CH)	135.1 (CH)	132.8 (CH)	132.8 (CH)	134.2 (CH)	46.3 (CH_2_)
5	197.1 (qC)	196.9 (qC)	203.8 (qC)	203.6 (qC)	202.9 (qC)	206.1 (qC)
6	133.5 (qC)	134.0 (qC)	55.5 (CH)	55.1 (CH)	53.5 (CH)	130.4 (qC)
7	144.5 (qC)	144.9 (qC)	135.0 (qC)	132.4 (qC)	122.7 (qC)	142.3 (qC)
8	127.2 (CH)	128.6 (CH)	131.7 (CH)	132.4 (CH)	143.1 (CH)	124.4 (CH)
9	134.8 (CH)	134.7 (CH)	132.3 (CH)	132.1 (CH)	132.0 (CH)	135.3 (CH)
10	19.1 (CH_3_)	19.4 (CH_3_)	119.9 (CH_2_)	120.5 (CH_2_)	128.0 (CH_2_)	19.1 (CH_3_)
11	56.6 (CH_2_)	54.0 (CH_2_)	91.7 (CH)	98.7 (CH)	166.9 (qC)	86.6 (CH)
OCH_3_				54.9 (CH_3_)		

a Recorded at 100 MHz in DMSO-*d_6_*.

**Table 3.** NMR data (DMSO-*d*_6_) for compound **7**.

**7**							
**C no.**	**δ_C_ (mult.)^a^**	**δ_H_ (mult., *J*/Hz)^b^**	**HMBC**	**C no.**	**δ_C_ (mult.)^a^**	**δ_H_ (mult., *J*/Hz)^b^**	**HMBC**
1	63.3 (CH_2_)	3.53 (brd, 8.0)	2, 3, 6, 11	1′a	66.6 (CH2)	3.70 (dd, 6.0, 10.8)	6′, 3′, 2′
2	79.1 (qC)			1′b		3.46 (dd, 6.0, 10.8)	6′, 3′, 2′
3	165.1 (CH)	7.55 (d, 5.6)	1, 2, 4, 5, 6,	2′	80.4 (qC)		
4	132.8 (CH)	6.31 (d, 5.6)	2, 3, 5, 6	3′	161.6 (CH)	7.37 (d, 6.0)	6′, 5′, 3′, 2′
5	203.6 (qC)			4′	134.9 (CH)	6.28 (d, 6.0)	6′, 5′, 3′, 2′
6	55.1 (CH)	3.48 (s)	2, 3, 4, 5, 7, 8	5′	196.7 (qC)		
7	132.6 (qC)			6′	135.2 (qC)		
8	132.8 (CH)	6.37 (d, 10.0)	6, 7, 9, 10, 11	7′	140.7 (qC)		
9	132.2 (CH)	6.59 (ddd, 10.0, 10.0, 17.2)	8	8′	126.9 (CH)	7.78 (dd, 1.4, 16.0)	11′, 7′, 10′
10a	120.3 (CH_2_)	5.30 (d, 17.2)	8, 9	9′	134.7 (CH)	6.41 (m)	8′, 10′
10b		5.26 (d, 10.0)	8, 9	10′	19.1 (CH_3_)	1.88 (dd, 1.4, 6.8)	8′, 9′
11	97.9 (CH)	5.08 (s)	1, 7, 8, 11′	11′a	62.7 (CH_2_)	4.86 (d, 10.4)	11, 7′, 6′, 8′
OH-2		5.95 (s)	1, 2, 3, 6	11′b		4.49 (d, 10.4)	11, 7′, 6′, 8′
				OH-1′		4.93 (t, 6.0)	1′, 2′
				OH-2′		5.80 (s)	6′, 2′, 1′

a Recorded at 100 MHz;

b Recorded at 400 MHz.

**Table 4.** Cytotoxic activity of compounds **1**–**13**.

**Compounds**	**Cell Line IC_50_ (ppm)**		
	**HCT116^a^**	**A431^b^**	**A549^c^**
**1**	3.2	6.1	6.4
**2**	3.0	6.4	4.8
**3**	5.2	7.5	>20
**4**	4.7	6.6	11.1
**5**	2.3	3.9	>20
**6**	6.8	15.3	15.9
**7**	2.8	9.4	12.8
**8**	1.8	3.1	3.5
**9/10**	3.1	3.6	>20
**11**	>20	>20	>20
**12**	2.4	3.3	>20
**13**	7.2	>20	>20

a HCT116: human colorectal carcinoma;

b A431: human epidermal carcinoma;

c A549: human lung cancer.
